# Neuron-specific analysis of histone modifications with post-mortem brains

**DOI:** 10.1038/s41598-020-60775-z

**Published:** 2020-02-28

**Authors:** Kagari Koshi-Mano, Tatsuo Mano, Maho Morishima, Shigeo Murayama, Akira Tamaoka, Shoji Tsuji, Tatsushi Toda, Atsushi Iwata

**Affiliations:** 1 Department of Neurology, Graduate School of Medicine, The University of Tokyo, 7-3-1 Hongo, Bunkyo-ku, Tokyo 113-8655 Japan; 2 Department of Neuropathology, Tokyo Metropolitan Geriatric Hospital, 35-2 Sakaecho, Itabashi, Tokyo 173-0015 Japan; 3 Department of Neurology, University of Tsukuba, 1-1-1 Tennodai, Tsukuba, Ibaraki 305-8575 Japan

**Keywords:** Molecular medicine, Neurology

## Abstract

Histone modifications govern chromatin structures and regulate gene expression to orchestrate cellular functions in the central nervous system, where neuronal cells are postmitotic and developmentally inactive, the functional and age-dependent changes also accumulate in the epigenetic states. Because the brain is composed of several types of cells, such as the neurons, glial cells, and vascular cells, the analysis of histone modifications using bulk brain tissue might obscure alterations specific to neuronal cells. Furthermore, among the various epigenetic traits, analysis of the genome-wide distribution of DNA methylation in the bulk brain is predominantly a reflection of DNA methylation of the non-neuronal cells, which may be a potential caveat of previous studies on neurodegenerative diseases using bulk brains. In this study, we established a method of neuron-specific ChIP-seq assay, which allows for the analysis of genome-wide distribution of histone modifications specifically in the neuronal cells derived from post-mortem brains. We successfully enriched neuronal information with high reproducibility and high signal-to-noise ratio. Our method will further facilitate the understanding of neurodegeneration.

## Introduction

Histone modification is a part of the epigenome that includes covalent post-translational methylation, acetylation, or ubiquitylation of histone proteins. These modifications co-operate with other epigenetic factors^1^ such as DNA methylation or non-coding RNA, to alter chromatin structures, orchestrate gene expression, and regulate cellular functions, including cell division, growth, and differentiation during the developmental process^2,3^. In the central nervous system, where mature neuronal cells are postmitotic and developmentally inactive, histone modifications play a key role in memory formation and learning process contributing to neuronal plasticity^4,5^. Aging is also associated with chromatin remodeling, and a better understanding of the phenomenon could be leveraged to induce a variety of responses to restore youthful functionalities in old tissues^6–8^. Furthermore, in neurodegenerative conditions, such as Alzheimer’s and Parkinson’s disease, profound effects on histone modifications are thought to reflect the pathogenic neurodegenerative processes^9,10^. As a result, histone modifications that exist in mature neuronal cells have a complex structure, *post hoc* modifications corresponding to physiological and/or pathological process, layered on a *priori* modification specific to neuronal cells. Thus, genome-wide profiles of histone modifications specific to neuronal cells can facilitate the elucidation of physiological mechanisms of the brain related to learning and memory, and pathomechanisms, where various life-long factors converge to cause neurodegeneration.

When analyzing histone modifications in brain samples, we must consider the fact that the brain is composed of several types of cells, including neuronal cells that directly contribute to learning and memory, glial cells that support neuronal activities or provoke inflammation, and vascular cells that deliver oxygen and nutrition to the brain. Each type of cell has its own specific histone modification corresponding to its developmental process, and subsequently acquires alterations in the modifications based on its physiological and pathological condition. Therefore, histone modification of the bulk brain derived from the cerebral cortex is a mixture of that of neuronal and non-neuronal origins. Considering that neurons comprise approximately 40%^11–13^ of all the cells in the cortex, bulk brain analysis is not representative of the neuronal epigenome. Thus, we hypothesized that the genome-wide profiles of histone modification in neuronal cells cannot be estimated by using bulk brain tissue, and this motivated us to develop a method for understanding the genome-wide profiles of histone modifications specific to neuronal cells.

Chromatin immunoprecipitation sequencing (ChIP-seq) is a method used to identify genome-wide profiles of histone modifications, where the genomic DNA that is wrapped around histone proteins is co-immunoprecipitated using a modification-specific anti-histone antibody to prepare libraries for next generation sequencing. For neuron-specific analysis, we applied fluorescence activated cell sorting (FACS)-based isolation of neuronal nuclei. Formerly, large number of cells was required for robust and reproducible ChIP-seq analysis and this used to be a major challenge for FACS isolation of neuronal nuclei where the number of the nuclei that could be isolated was limited. Especially, for studying neurodegenerative conditions where post-mortem brain samples are used and the amount of sample available for the assay is limited, the number of the nuclei required for the assay should ideally be low. The condition of the sample used in the assays is also critical for reproducibility because post-mortem brain samples are inevitably affected by the post-mortem time to autopsy and subsequent freeze-thaw processes. To overcome these issues, we optimized each step of the FACS and ChIP-seq that enabled multiple genome-wide histone modification analyses. Here, we demonstrate that neuron-specific histone modifications are completely different from non-neuron-specific, and bulk brain histone modifications, emphasizing the importance of neuronal isolation for post-mortem brain epigenome analysis.

## Results

### Optimization of crosslinking methods

**Figure 1:**
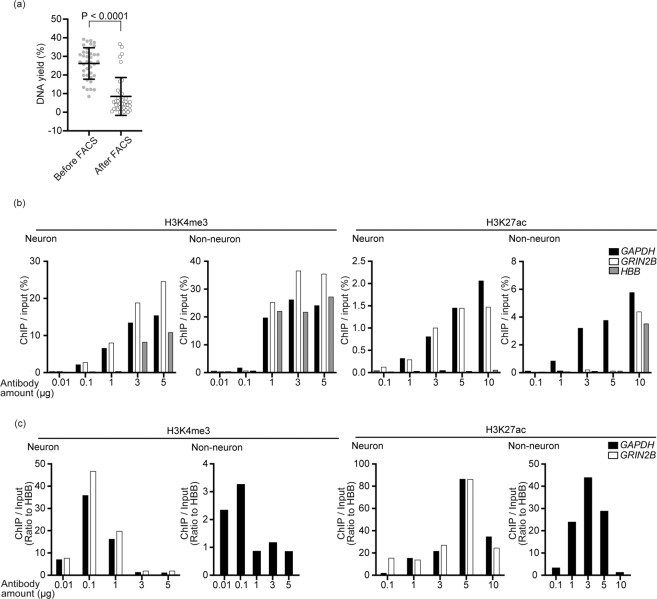
Optimization of fixation and immunoprecipitation. (**a**) The relationship between the DNA yield and the duration of crosslinking. The DNA yield was calculated based on the assumption that the amount of genomic DNA in a single human cell was 6.6 pg^35^. Dark gray dots represent data before FACS, the light gray dots represent data after FACS, and the black bar represent mean ± SD. The statistical significance was determined by *t*-test. N = 38. (**b**,**c**) The relationship between the antibody amount and fold enrichment of the house-keeping gene (*GAPDH*), a neuronally expressed gene (*GRIN2B*) and non-neuronally expressed gene (*HBB*). Fold enrichment was calculated using qPCR of enriched DNA fragment as ChIP/Input (%) (**b**) or its ratio to *HBB* (**c**).

**Figure 2:**
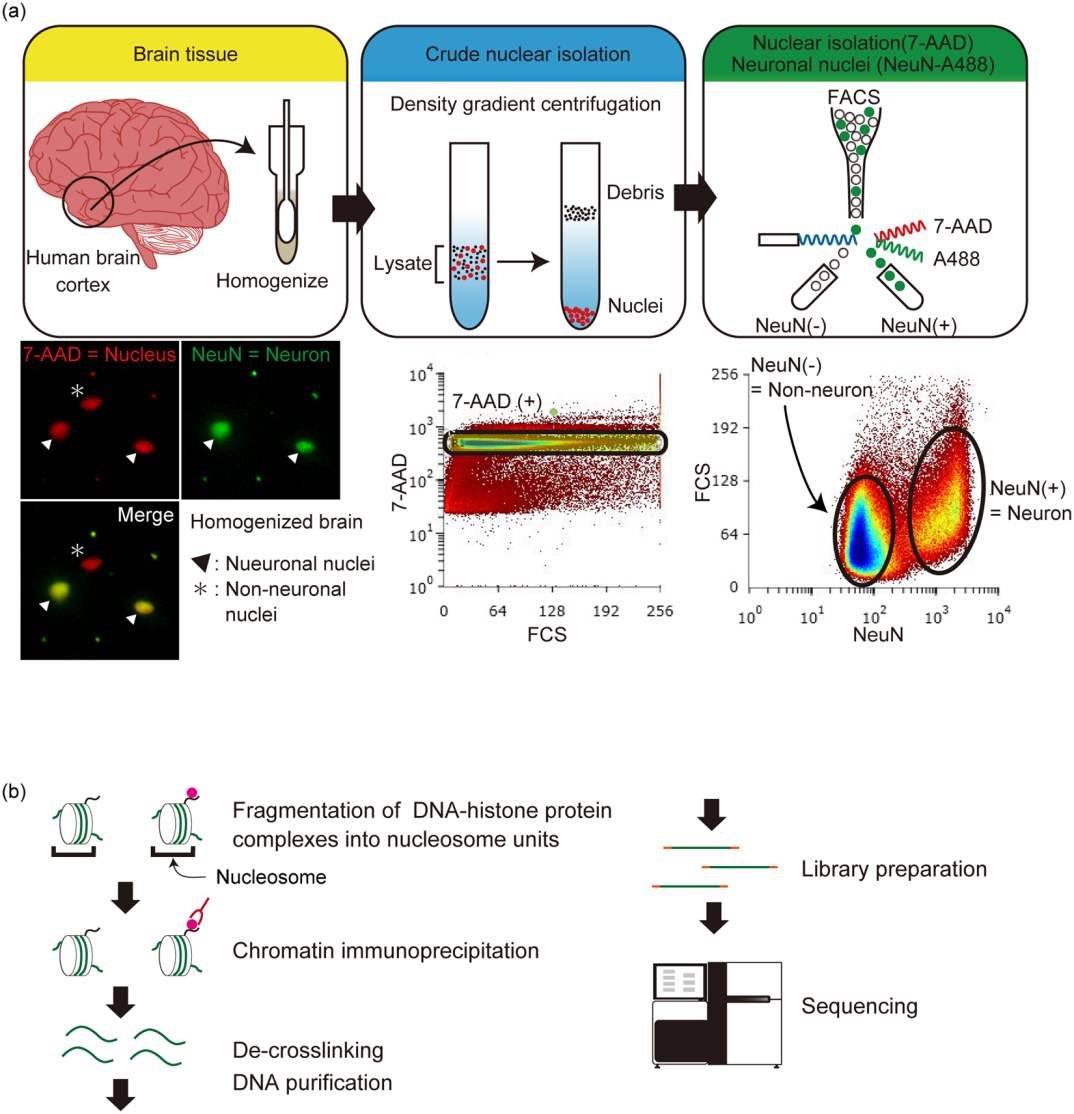
Schematic illustration of neuronal nuclei isolation and chromatin immunoprecipitation. (**a**) Isolation of neuronal nuclei using FACS. Upper panel shows the schematic process of each step, and lower panels show representative data. Brain samples were homogenized and subjected to density gradient centrifugation to obtain crude nuclear isolates. All the nuclei were stained with 7-AAD, and separation of neuronal and non-neuronal nuclei was performed by Alexa488-conjugated anti-NeuN antibody. FACS separation was performed. The lower panels are immunofluorescence images of neuronal and non-neuronal nuclei and neuronal/non-neuronal nuclei isolation using FACS. (**b**) Schematic diagram of ChIP. The obtained nuclei were sonicated to fragment the genomic DNA into nucleosome units. The nucleosomes with the target histone modifications were captured using an antibody against the target modification. The captured DNA was purified and subjected to library preparation and next generation sequencing.

The first step in the ChIP assay is the crosslinking of the nucleosome, which is composed of genomic DNA wrapped around histone proteins, and uniform reaction across the tissue is essential for reproducibility^14^. Generally, fixation in the early steps ensures optimum crosslinking. However, when using tissue sample, fixing brain tissue *en block* has a serious disadvantage in that the surface of the brain may be fixed more than its inside potentially leading to uneven ChIP-seq assay. Given that the separation of neuronal nuclei from non-neuronal ones using FACS is required to achieve specificity in neuronal ChIP-seq^15^, optimization of this step is crucial. Therefore, we tested two different time points for fixing the nucleosome complexes; (1) immediately after homogenization of the frozen brain or (2) after FACS. All the brains were obtained from the patients without any pathological conditions in the brain. When compared to the yield of genomic DNA extracted before DNA fragmentation, the yield was higher and more reproducible when the nuclei were fixed immediately after homogenization (Mean ± SD: 26.2 ± 8.4% vs 8.5 ± 10.2%) (Fig. 1a). We speculated that this was because before fixation the bare nuclear membrane can be easily fragmented during FACS. With this method, we obtained 47.4 ± 19.3 neuronal nuclei and 78.8 ± 30.1 non-neuronal nuclei from 100 mg of the brain tissue (Fig. S1a). Separation of neuronal and non-neuronal nuclei confirmed by immunofluorescence staining and western blotting (Figs. 2a and S1b). On the completion of nuclear isolation, the neuronal or non-neuronal nuclei were subjected to sonication to fragment the genomic DNA into lengths of 150–250 bp according to the standard sonication protocol, such that the genomic DNA was fragmented into single nucleosome units (Fig. S2).

### Optimization of ChIP assay

We then optimized the amount of antibodies used in the immunoprecipitation of two representative histone modifications, H3K4me3 and H3K27ac, that are positively correlated with gene expressions^16^. In general, the amount of DNA fragments captured by antibodies depends on the amount of antibodies used for the immunoprecipitation, however, excessive amounts of antibodies could potentially result in non-specific binding and increase undesirable signal in regions without target modifications. On the other hand, a low yield of DNA fragments requires more PCR cycles resulting in reduced library complexity and skewing of the library. Thus, optimization of the amount of antibody is crucial to obtain a good quality library with high sensitivity and specificity. To validate the yield and specificity, we performed qPCR with the immunoprecipitated DNA fragments. To assess the specificity of our neuronal and non-neuronal ChIP, we chose three genomic regions for qPCR as below, where are supposed to be enriched with the histone modifications analyzed in this study according to their expressions. ChIP reaction was performed using 2 × 10^6^ nuclei in the reaction volume of 1 mL. We measured the fold enrichment of TSS regions of *GAPDH* (endogenous positive control), *GRIN2B* (neuron-specific marker) and *HBB* (negative control that is not expressed in the central nervous system)^17^. Along with the increasing amounts of antibodies, the fold enrichment of *GAPDH*, which is the universally expressed gene in all tissues, was enhanced (Fig. 1b), however, the signal from the negative control gene *HBB*, that is not expressed in the central nervous system, also increased thus lowering the signal to noise ratio (Fig. 1c). The TSS region of *GRIN2B* was enriched only in neuronal samples, which was consistent with specific expression of *GRIN2B* in neuronal cells. Based on these data, we determined that the optimal amount of antibody per assay is 0.1 μg for anti-H3K4me3 and 5 μg for anti-H3K27ac antibody. To validate the above-established ChIP method, we performed ChIP using more brain samples and qPCR to measure fold enrichment of the above regions, demonstrating the robustness of our method. Furthermore, we performed qPCR with the ChIP samples in other genomic regions including *SYN3* and *BDNF*, neuron-specific genes, and *ERMN* and *OLIG2*, non-neuronal genes. As expected, *SYN3* and *BDNF* were enriched only in neuronal ChIP samples, and *ERMN* and *OLIG2* in non-neuronal ChIP samples. Taken together, we could confirm neuronal-enrichment in our neuron-specific ChIP in the local genomic regions, thus, we moved on to the genome wide validation of our method.

**Figure 3:**
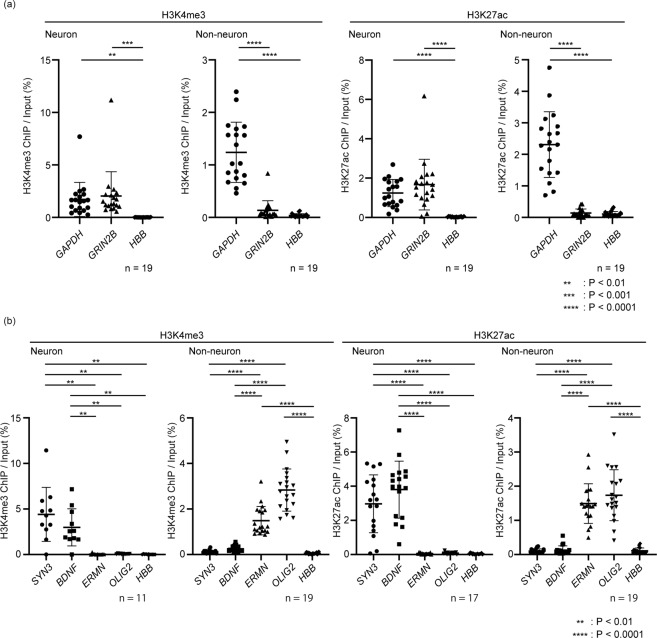
quantitative PCR of neuronal and non-neuronal ChIP samples. (**a**) To validate neuronal and non-neuronal specificity of ChIP samples, samples were subjected to qPCR for *GAPDH, GRIN2B* and *HBB* which were also used in the optimization step (Fig. 1). (**b**) qPCR was also performed for *SYN3* and BDNF, neuronal regions, and *ERMN* and *OLIG2*, non-neuronal regions. Fold enrichment was calculated using qPCR of enriched DNA fragment as ChIP/Input (%). The black bar represents mean ± SD. Statistical significance was determined by one-way ANOVA with post-hoc Turkey (H3K4me3 ChIP using neurons) and Brown-Forsythe ANOVA with post-hoc Dunnett’s correction (the others). The number of samples is shown in each panel. **P < 0.01, ***P < 0.001, ***P < 0.0001.

**Figure 4:**
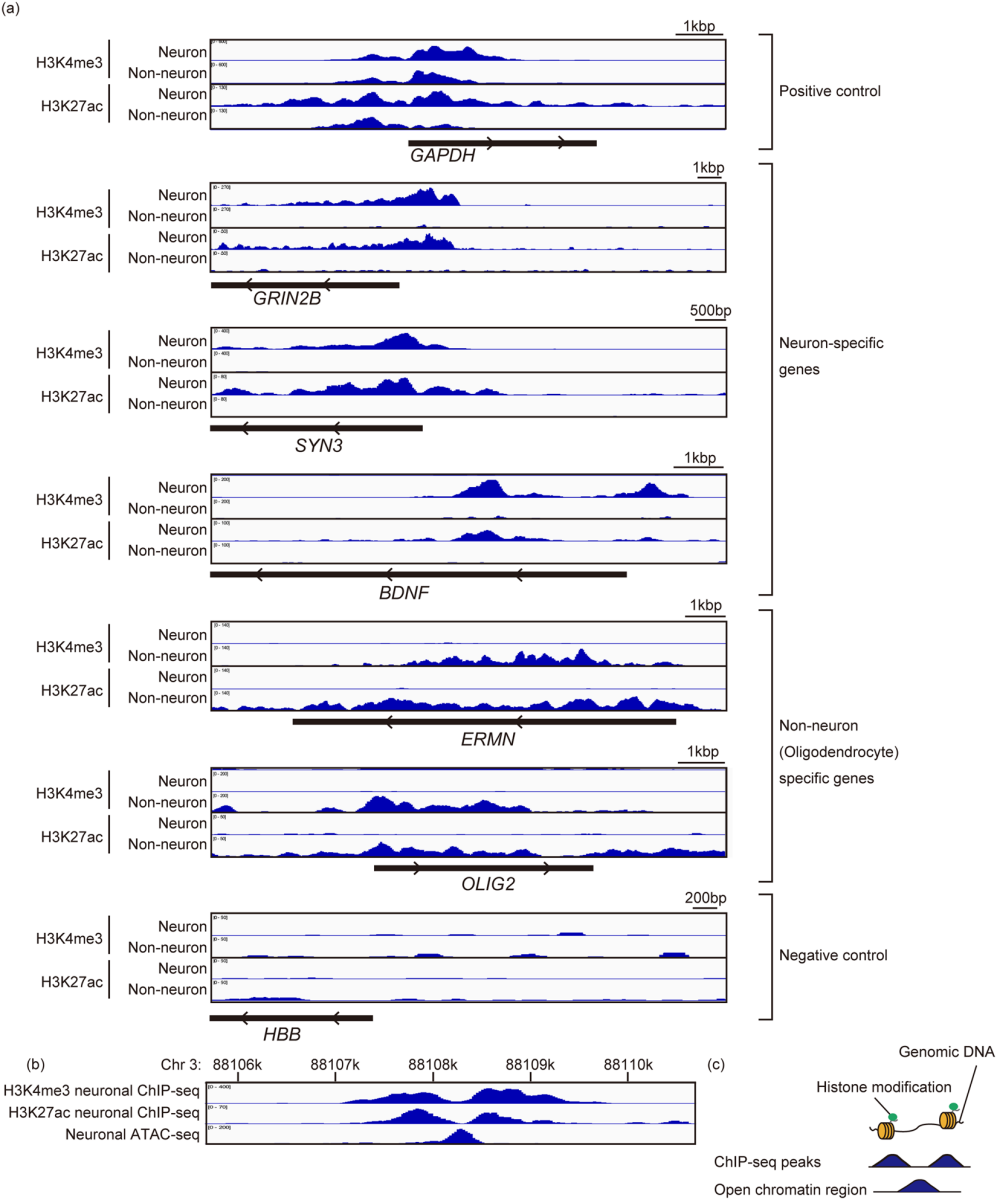
The distribution of mapped reads. (**a**) The distribution of mapped reads around TSS of *GAPDH*, *GRIN2B* and *HBB*. ChIP-seq peak of H3K4me3 and H3K27ac in neuronal and non-neuronal cells is displayed on the IGV browser. Black bars under the distribution of the mapped reads show the position and orientation of genes. (**b**) Representative distribution of ChIP-seq peaks and ATAC-seq peak^18^. The peaks of H3K4me3 ChIP-seq, H3K27ac ChIP-seq were consistent with ATAC-seq peak. (**c**) Scheme illustrating the relationship with ChIP-seq peaks and chromatin structure. ChIP-seq peaks represent the genomic region wrapping around the histones with specific modifications, and ATAC-seq peaks represent the genomic region free from protein bindings, thus, ATAC-seq peak generally exist between the ChIP-seq peaks.

The DNA fragments were subjected to library preparation. To check the quality of the library, the fold enrichment of the three genes, *GAPDH*, *GRIN2B*, and *HBB*, was validated just before sequencing, which demonstrated that the library preparation process did not change the enrichment patterns (Fig. S3). Consistent with the result of qPCR in Fig. 3, the distributions of the mapped reads of the genes analyzed showed distinctive patterns according to neuronal or non-neuronal origins (Fig. 4a). Furthermore, the peaks detected in H3K27ac and H3K4me3 ChIP-seq in neuronal cells were well overlapped with open-chromatin regions defied by the publicly available ATAC-seq in neuronal cells^18^, suggesting the peaks of neuron-specific ChIP-seq were indeed associated with transcriptionally active regions (Fig. 4b,c).

### Genome-wide profiles of neuronal and non-neuronal ChIP-seq

**Figure 5:**
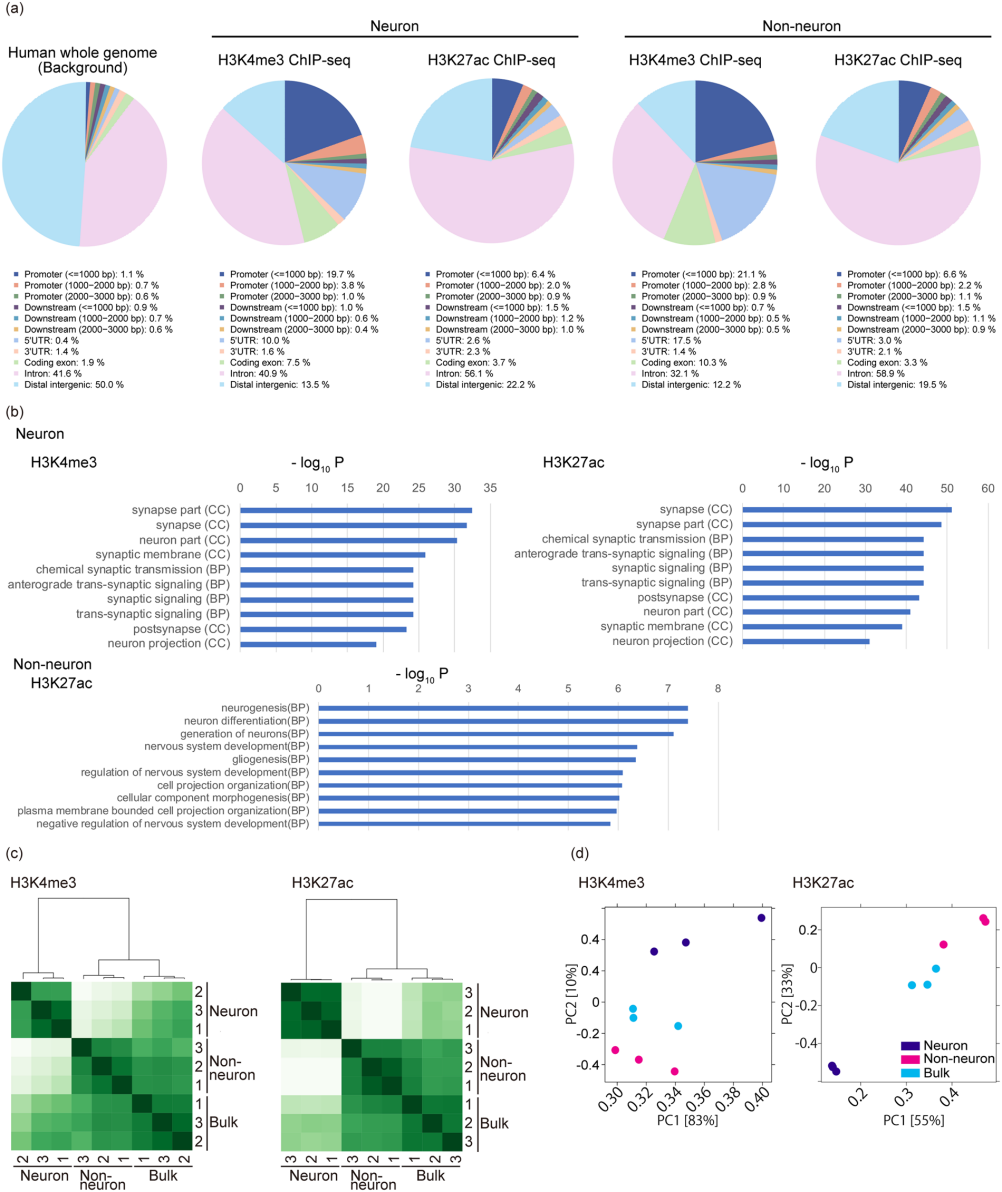
The genome-wide profiles of H3K4me3 and H3K27ac ChIP-seq peaks in neuronal cells, non-neuronal cells and bulk brain. (**a**) Genomic annotation of H3K4me3 and H3K27ac profiles in neuronal and non-neuronal cells. Each ChIP-seq peak was analyzed by CEAS. (**b**) The top 10 significantly enriched GO terms (y-axis) and their p-values (x-axis) of differentially modified genes. Only the terms with statistical significance (P < 0.01) are shown, such that no terms are shown for H3K4me3 ChIP-seq of non-neuronal samples. (**c**) Clustering analysis of ChIP-seq peak in neuronal, non-neuronal cells and bulk brain. (**d**) Principal component analysis of ChIP-seq peak in neuronal, non-neuronal cells and bulk brain.

Next, we analyzed the genome-wide profiles of the histone modifications. Compared to the background profile of the human whole genome, H3K4me3 modifications in both the neuronal and non-neuronal samples were enriched in the active promoter regions (2.4% vs 24.5% and 24.8%, respectively), and H3K27ac was also enriched around TSS region, suggesting successful enrichment of each histone mark feature (Fig. 5a)^19^. Gene ontology analysis also supported the enrichment of neuronal ontology in H3K4me3 and H3K27ac ChIP-seq of neuronal samples. On the other hand, non-neuronal samples showed less or no significant enrichment in H3K27ac or H3K4me3, respectively (Fig. 5b). In the clustering and principal component analysis, neuronal, non-neuronal and bulk samples were well clustered within each cell type, but the neuronal group formed a distinctive cluster from other 2 groups. The genome-wide distribution of histone modifications obtained from the bulk samples was similar to that obtained from the non-neuronal samples, but was distinctive from the neuronal samples (Fig. 5c,d), which can be attributed to the fact that the ratio of the amount of neurons to non-neurons, even in the cortex, was approximately 2:3 (Fig. S1a). This suggests that the isolation of neuronal nuclei is essential when analyzing neuronal histone modifications, and is not possible using bulk brain tissue analysis. These data support our hypothesis that ChIP-seq of bulk samples does not necessarily reflect neuronal histone modification, but rather reflects that of the non-neuronal cells. Finally, we also confirmed high reproducibility between technical replicates (the Pearson’s coefficient 0.99) (Fig. S4), supporting the robustness of our method described above.

## Discussion

Here, we established a method for neuron-specific ChIP-seq analysis. Optimization of the fixation process enhanced the yield and reproducibility of DNA extraction, and optimal amount of antibody in immunoprecipitation step increased the ChIP efficiency and minimized non-specific binding. The genome-wide profiles of histone modifications in neuronal cells have distinctive patterns compared to that of the bulk brain or non-neuronal cells, suggesting that the genome-wide profiles of histone modification in neuronal cells cannot be estimated using that of the bulk brain.

Unlike in the DNA methylome analysis, where the covalent binding of the methyl group to cytosine retains the modification during the assay, ChIP-seq requires fixation to maintain the interaction between the DNA and the histone proteins because the DNA only wraps around the histone proteins without any covalent binding. As discussed earlier, fixation immediately following homogenization improves the yield of genomic DNA compared to fixation after FACS. This may be because the FACS process impairs the nuclear membrane, thus allowing genomic DNA to leak from the nucleus even with nuclear stabilization with calcium and magnesium supplementation.

The histone modification profile of the neuronal cells was distinctive from those of the bulk brain and non-neuronal cells. This result was consistent with DNA methylation study, where DNA methylation in the bulk brain was similar to non-neuronal cells, not to neuronal cells^20^. This can be attributed to the fact that the majority of cells comprising the brain are glial cells that have distinctive epigenomic feature compared to neuronal cells^21^. ChIP-seq.^9^ studies using bulk brains for Alzheimer’s disease have shown that some of the differentially modified regions are associated with risk loci identified by genome-wide association study (GWAS). GWAS analyzes genomic sequences that are identical throughout the body and has no specificity to its derived cell type. Therefore, these studies suggest the possibility that epigenetic changes in the neuronal cells were obscured in the bulk analysis and epigenetic analysis using bulk brain can only extract the information that cell-type independent GWAS can identify. On the other hand, neuron-specific DNA methylation analysis of post-mortem brains from Alzheimer’s disease patients could help to identify a novel pathomechanism occurring exclusively in neuronal cells^15^. Chromatin accessibility analysis with the Assay for Transposase Accessible Chromatin followed by sequencing (ATAC-seq) in neuronal cells from schizophrenia also provided a novel single nucleotide polymorphism (SNP) with biological relevance^22^. These reports suggest the importance of neuron-specific epigenetic analysis. Neuron-specific ChIP-seq can potentially shed light on novel neuronal phenomena and elucidate molecular events occurring in neuronal cells.

As for genomic annotations of ChIP-seq enrichment, the promoter regions were enriched in H3K4me3 (Fig. 4a), which was consistent with previous reports that H3K4me3 was enriched in active promoters. Notably, compared to H3K27ac, the 5′ UTR was also enriched in H3K4me3 ChIP-seq (in neuronal cells, 10% vs 2.6% for H3K4me3 and H3K27ac, respectively). A recent study has shown that METTL3 is recruited to TSS characterized by H3K4me3 modifications^23^. METTL3 forms an N6-methyltransferase complex that co-transcriptionally deposits N6-methyladenosine (m^6^A) to RNA and regulates various biological processes^24,25^. In neuronal cells, approximately half of expressed mRNAs are m^6^A modified, and decreased m^6^A impairs neurogenesis and neuronal functions, supporting the importance of m^6^A deposition by METTL3^26^. Our data imply that the genomic structure of 5′UTR regulated by H3K4me3 might be associated with neuronal functions through METTL3 recruitment.

Neuronal cells extend projections called neurites to connect with each other at synapsis to form functional networks, which is a distinctive feature unlike in other systemic organs where their functions are dependent on the cell number. Neuronal network is the structural basis for memory and learning process, and previous reports have demonstrated that histone modifications play a pivotal role^27–31^. Consistent with these reports, the two histone modifications analyzed in this study in neuronal cells showed prominent enrichment of the ontology terms associated with synapse and neurite projection. Non-neuronal cells also showed enrichment of the terms associated with the environment of the central nervous system maintaining neuronal and glial functions. However, their enrichment was much weaker than that of neuronal cells, which may be attributed to the fact that non-neuronal cells are a heterogeneous cell population and includes astrocytes, oligodendrocytes, and vascular cells.

Neuron-specific ChIP-seq is advantageous in that it allows us to summarize the genomic profile of histone modifications in many neuronal cells and extract common and representative changes among them. However, this assay abolishes the heterogeneity of neuronal cells in the summarization process, which in some cases could be a disadvantage. For example, only a part of the excitatory neurons is activated in physiological conditions. Neuronal cells can be categorized into several subpopulations and some intact cells remain even in the advanced stages of Alzheimer’s disease. The recently developed single-cell ChIP-seq technique overcomes such heterogeneity issues to successfully identify even sub-populations within a supposedly single-type of cell population^32^. However, technical limitations such as insufficient coverage narrows the detectable range to the genes with higher expression, and still prevent discrimination of phenotype-specific alterations from variations within cell types. Cell-type specific ChIP-seq collects histone modifications data from each cell to converge the data and minimize variations within cell types, and thus can predict the transcriptional changes in genes with low expression that is more suitable for the study of neurodegenerative disorders.

In summary, we established a method for neuron-specific ChIP-seq using post-mortem brain samples. The optimization of the fixation and immunoprecipitation conditions enables ChIP-seq to be highly specific to neuronal cells, with enhanced enrichment and reproducibility. Neuron-specific ChIP-seq will expand our understanding of neuronal plasticity and the neurodegenerative process.

## Materials and Methods

### Human brain samples

This research was approved by the ethics committee of the University of Tokyo (approval #2183-17). All human samples were used in accordance with the principles of the Declaration of Helsinki.

We collected postmortem brains with written consent from the patients’ families and maintained them at −80 °C until use. The brain samples from 22 normal subjects were obtained from The University of Tokyo, Tokyo Metropolitan Geriatric Hospital brain bank and Tsukuba University, of which 3 samples were subjected to ChIP-seq and 19 samples were subjected to ChIP with qPCR analysis. Trained neuropathologists made a pathological diagnosis of the brains and confirmed no pathological changes in the brain. The detailed demographics of the brain samples are shown in Supplementary Tables 1 and 2.

### Neuronal/Non-neuronal nuclei isolations

Human brain cortex were dissected from the temporal or parietal lobes (500 mg – 1,000 mg per assay), and homogenized using a Teflon homogenizer (AS ONE, Osaka, Japan) in nuclear buffer (10 mM Tris-HCl pH 8.0, 0.32 M sucrose, 5 mM CaCl_2_, 3 mM MgAc_2_, 0.1 mM EDTA and 0.1% (v/v) Triton X-100) supplemented with a protease inhibitor cocktail (Roche, Basel, Switzerland). Brain homogenates were subjected to a Percoll (GE Healthcare, Pittsburgh, PA, USA) step gradient on four-layers (12%, 17.4%, 26%, and 35%), centrifuged at 16,000 × *g* for 30 min at 4 °C, and the nuclei were collected from the 35% Percoll fraction. The isolated nuclei were blocked with 1.5% bovine serum albumin (BSA) and stained with Alexa Flour 488-conjugated anti-NeuN antibody (Millipore, Darmstadt, Germany, 1:500, clone A60, AB_2149209) and 15 μg/mL of 7-amino-actinomycin D (7-AAD, BioLegend, San Diego, USA) overnight at 4 °C, and subjected to fluorescence-activated cell sorting (FACS). The nuclei were first gated on the basis of 7-AAD intensity, where all the nuclei were stained equally, and then subjected to separation of the neuronal and non-neuronal nuclei by NeuN-A488 intensity (Fig. 2). To stabilize the sorted nuclei, MgAc_2_ and CaCl_2_ solutions were added to post-FACS nuclei samples (final concentrations: 0.3 M sucrose, 2.4 mM MgAce_2_, 24 mM CaCl_2_)^33^. To crosslink the DNA and histone proteins, the nuclei were fixed immediately after homogenization or nuclei sorting depending on the methods.

### Western blotting

Isolated nuclei were resuspended with 1 × lithium dodecyl sulfate (LDS) buffer (Invitrogen, Carlsbad, CA, USA) and 1% of 2-Mercaptoethanol and incubated at 60 °C with for 15 min. Samples were resolved on SDS-PAGE, and the separated proteins were transferred to polyvinylidene difluoride (PVDF) membranes using a Trans-Blot Turbo Blotting System and Trans-Blot Turbo Transfer Pack (Bio-Rad, Hercules, CA, USA). Non-specific binding was blocked with EzBlock CAS (ATTO Corporation, Tokyo, Japan) for 60 min at 25 °C. Membranes were then incubated with primary antibodies in CanGetSignal Solution I (TOYOBO, Osaka, Japan) for 2 h at 25 °C or overnight at 4 °C. Anti-NeuN (A60, Millipore Cat# A60, RRID:AB_2314889, 1:1000) and anti-Olig2 (Millipore Cat# AB9610, RRID:AB_570666, 1:1000) were used as primary antibodies. Membranes were washed with TBST for 10 min three times, and then incubated with secondary horseradish peroxidase-conjugated antibodies (GE Healthcare, Little Chalfont, UK), diluted 1:2000 in TBST for 1 h at 25 °C. After membranes were washed with Tris-buffered saline (TBS-T) for 10 min three times, they were visualized by EzWestLumi Plus (ATTO). Images were captured by LuminoGraph I (ATTO), and quantified using Image J software (http://imagej.nih.gov/ij/).

### Fragmentation of DNA-histone protein complexes into nucleosome units

The isolated nuclei were centrifuged at 4,000 rpm 4 °C for 5 min, resuspended in pre-chilled lysis buffer 1 (50 mM HEPES-KOH pH 7.5, 140 mM NaCl, 1 mM EDTA pH 8.0, 10% w/v glycerol, 0.5% w/v NP-40, 0.25% w/v Triton X-100), and incubated at 4 °C for 10 min. Subsequently, the nuclei were resuspended in pre-chilled lysis buffer 2 (10 mM Tris-HCl pH 8.0, 200 mM NaCl, 1 mM EDTA pH 8.0, 0.5 mM EGTA pH 8.0) and lysis buffer 3 (50 mM Tris-HCl pH 8.0, 1% SDS, 10 mM EDTA pH 8.0), sequentially, according to a previously published protocol^34^. The nuclei were sonicated using a Focused-ultrasonicator (Covaris E220 (Covaris, Woburn, MA, USA), duty: 5.0%, Intensity/peak incident power (PIP): 105, Cycles per burst: 200, Treatment time: 1200 seconds) with its genomic DNA becoming 150–250 bp in length (Fig. S1). The fragmented DNA solution was centrifuged at 16,000 × *g* for 5 min at 4 °C to remove the debris, and the supernatant was aliquoted into the corresponding amounts (2 × 10^6^ nuclei per immunoprecipitation and 2 × 10^5^ nuclei per input sample). The fragmented DNA samples were frozen at −80 °C until further processing.

### Chromatin immunoprecipitation and DNA purification

Prior to immunoprecipitation, the lysates corresponding to 2×10^6^ nuclei were diluted to 1 mL volume with the ChIP dilution buffer (16.7 mM Tris-HCl pH 8.0, 0.01% SDS, 1.1% Triton X-100, 1.2 mM EDTA pH 8.0, 167 mM NaCl). Anti-histone antibodies (anti-H3K4me3 antibody, abcam, #8580; anti-H3K27ac antibody, Active motif, #39133) were added to the samples, and incubated at 4 °C for 4 hours. Then, 40 μg of pre-washed magnetic beads (Dynabeads Protein G for H3K27ac or Dynabeads Protein A (Life Technologies) for H3K4me3) were added to capture antibody-histone complexes and incubated at 4 °C for 1 hour. The beads were then washed with 1 mL of pre-chilled Low salt buffer (20 mM Tris-HCl pH 8.0, 0.1% SDS, 1% w/v Triton X-100, 2 mM EDTA pH 8.0, 150 mM NaCl) 5 times followed by washing in 1 mL of pre-chilled High salt buffer (20 mM Tris-HCl pH 8.0, 0.1% SDS, 1% w/v Triton X-100, 2 mM EDTA pH 8.0, 500 mM NaCl) 3 times. The immunoprecipitated nucleosome complexes were eluted with 200 μL of ChIP Elution Buffer (10 mM Tris-HCl pH 8.0, 300 mM NaCl, 5 mM EDTA pH 8.0, 1% SDS) for 15 min at room temperature, and eluted samples were incubated at 65 °C overnight for de-crosslinking. The extracted DNA fragments were purified using a standard phenol/chloroform/isoamyl alcohol protocol.

### Quantitative PCR analysis

Quantitative real-time polymerase chain reactions (RT-qPCR) were performed to assess the enrichment of neuron-specific and histone modification-specific genome regions. qPCR primers were designed for the genome regions around the transcription start site (TSS) of *GAPDH* as housekeeping gene, *HBB* as a gene with low expression in the brain, *GRIN2B*, *SYN3* and *BDNF* as genes with high expression only in neuronal cells, and *ERMN* and *OLIG2* as genes with high expression only in non-neuronal cells (Table S3). qPCR was performed using PowerUp SYBR Green Master Mix (Thermo Fisher Scientific, CA, USA) with the Applied Biosystems 7900 Fast Time PCR system. The enrichments were calculated using the percent input method that is the signals obtained from the ChIP samples are divided by the signals obtained from the input samples.

### Library preparation and NGS sequencing

Input and immunoprecipitated DNA samples were subjected to end repair, A-tailing, adapter ligation, and amplification using KAPA Hyper Prep kit (KAPA Biosystems, Cape Town, South Africa) according to the manufacturer’s instructions. The library thus obtained was cleaned up with Agencourt AMPure XP (Beckman Coulter, Fullerton, CA, USA) and quantified using qPCR with KAPA Library Quantification kit (KAPA Biosystems) prior to sequencing. The libraries for technical replicates were sequenced on MiSeq (Illumina, San Diego, CA, USA) to obtain 20 million paired end reads (75 base pair), and libraries for neuron- and non-neuron specific ChIP-seq were sequenced on Hi-Seq2500 (Illumina) to obtain 40 million paired end reads (100 base pair).

### Data processing

The initial quality control and adaptor trimming were performed using Trimmomatic v.0.36 with standard parameters. The reads were mapped to the reference human genome (hg19) using Bowtie2 v.2.3.4 and subjected to peak calling using MACS2 v.2.1.1 with a q-value threshold of 0.01. The distribution of the mapped reads over the genome features was analyzed using CEAS v.1.0.2 for neurons. The differential binding and gene ontology analysis were performed using R 3.4.3/Bioconductor v.3.6 packages DiffBind v.2.6.6 and ChIPPeakAnno v.3.6.5, respectively. The reproducibility of the replicates of neuron-specific ChIP-seq was assessed using deeptools v.3.2.1. ATAC-seq (Assay for Transposase-Accessible Chromatin using sequencing) using the pre-frontal cortex was analyzed with publicly available data^18^.

## Supplementary information

Supplementary Information.
