# Supplementary material for: Neuron-specific analysis of histone modifications with post-mortem brains
Source: Sci Rep. 2020 Feb 28;10:3767. doi: 10.1038/s41598-020-60775-z (PMC7048733; doi:10.1038/s41598-020-60775-z)
Supplement: Supplementary file 1 — Supplementary Information. [file 41598_2020_60775_MOESM1_ESM.docx]

Supplementary Information

Neuron-specific Analysis of histone modifications with post-mortem brains

Kagari Koshi-Mano, Tatsuo Mano, Maho Morishima, Shigeo Murayama, Akira Tamaoka, Shoji Tsuji, Tatsushi Toda, Atsushi Iwata

**List of Figures**

**Supplementary Figure S1.** Neuronal and non-neuronal nuclei in human brain cortex

**Supplementary Figure S2.** The distribution of DNA fragment length

**Supplementary Figure S3.** Validation of of ChIP-seq library

**Supplementary Figure S4.** Assessment of neuron-specific ChIP-seq reproducibility

**Supplementary Figure S5.** Uncropped Western blots of Supplementary Figure 1a

**List of Tables**

## **Supplementary Table S1.** Demographic features of the brain samples for ChIP-seq

## **Supplementary Table S2.** Demographic features of the brain samples for qPCR

## **Supplementary Table S3.** The sequence of qPCR primers

## **
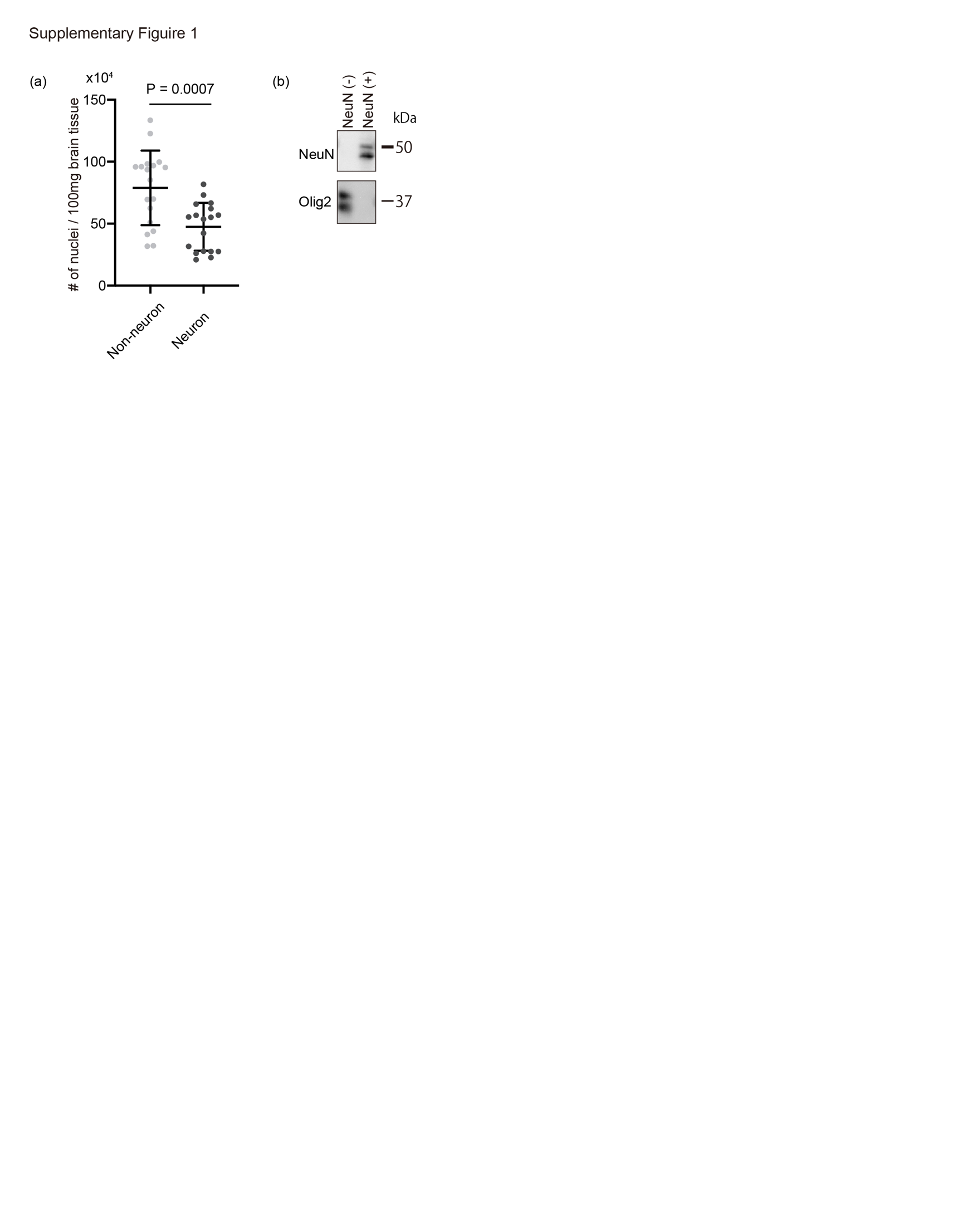
**

## **Supplementary Figure S1.** Neuronal and non-neuronal nuclei in human brain cortex

(a) The numbers of neuronal and non-neuronal nuclei obtained from 100 mg of brain tissue were measured by FACS. The statistical significance was determined by t-test. N = 18. Light gray dots represent data corresponding to non-neurons and dark gray dots represent data corresponding to neurons. (b) Neuronal and non-neuronal marker expression in the sorted nuclei. NeuN and Olig2 were representative markers for neuronal and non-neuronal neuclei.

*
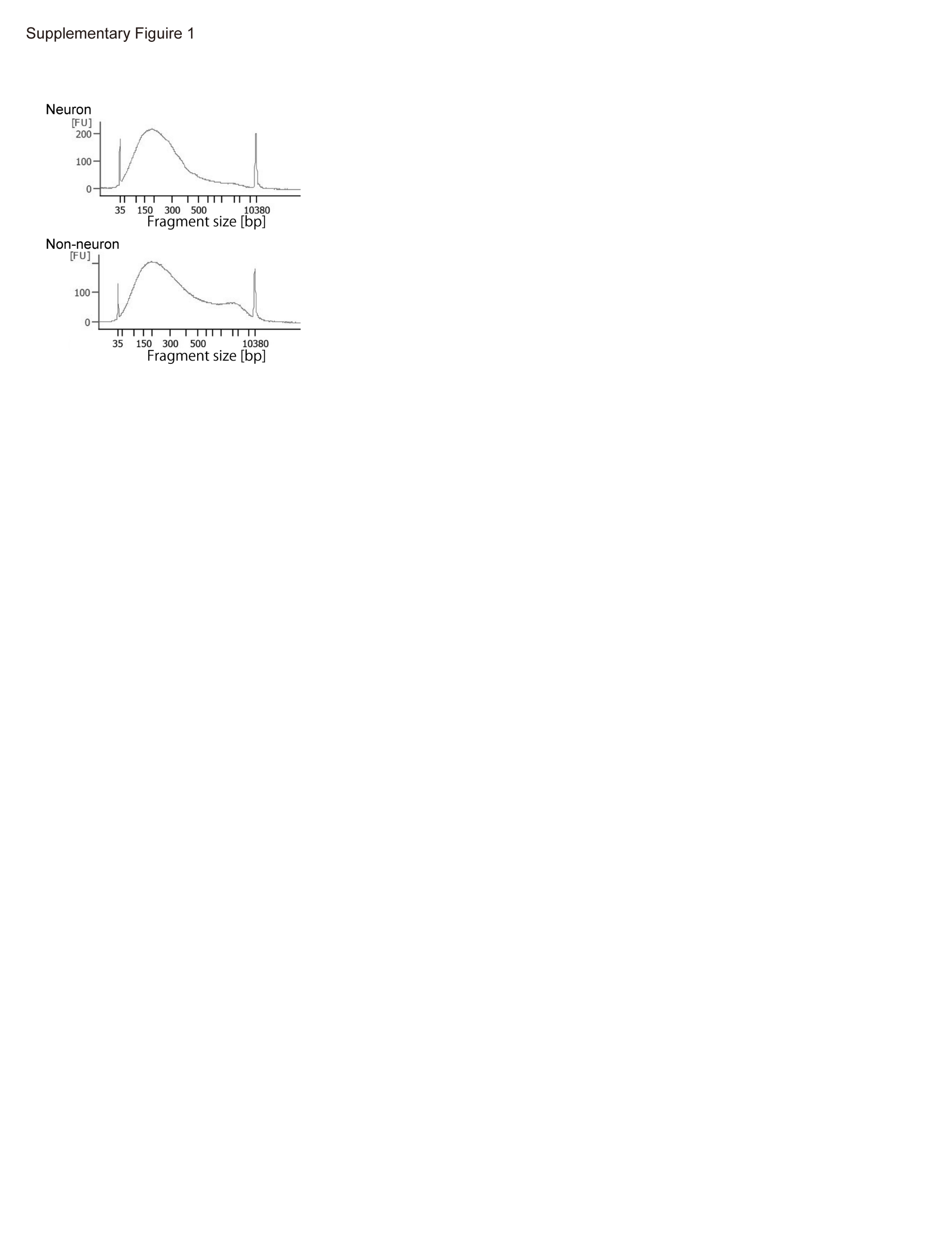
*

## **Supplementary Figure S2.** The distribution of DNA fragment length

Representative images of DNA fragmentation analyses. FACS-sorted nuclei were sonicated, and the fragmented genomic DNA was eluted. The lengths of the DNA fragments derived from the neuronal and non-neuronal cells were measured. The peaks on the two sides are size markers corresponding to 35 bp and 10,380 bp.

## **
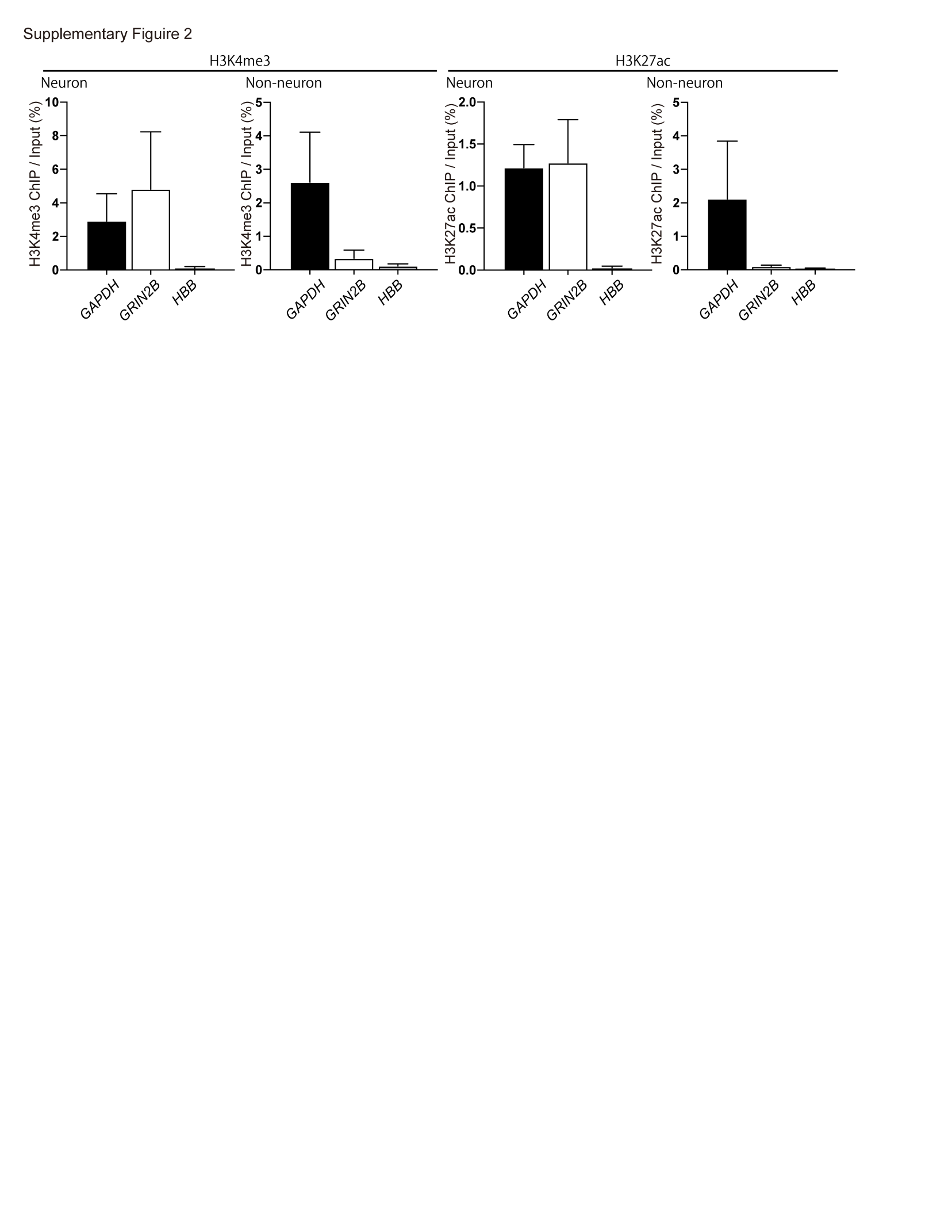
Supplementary Figure S3.** Validation of of ChIP-seq library

To validate the quality of ChIP-seq library, the libraries were subjected to qPCR for *GAPDH*, *GRIN2B,* and *HBB* just prior to sequencing. The fold-enrichment was calculated as ChIP / Input (%). Representative results are shown from a total n = 3 samples. The black bar represents mean ± SD.


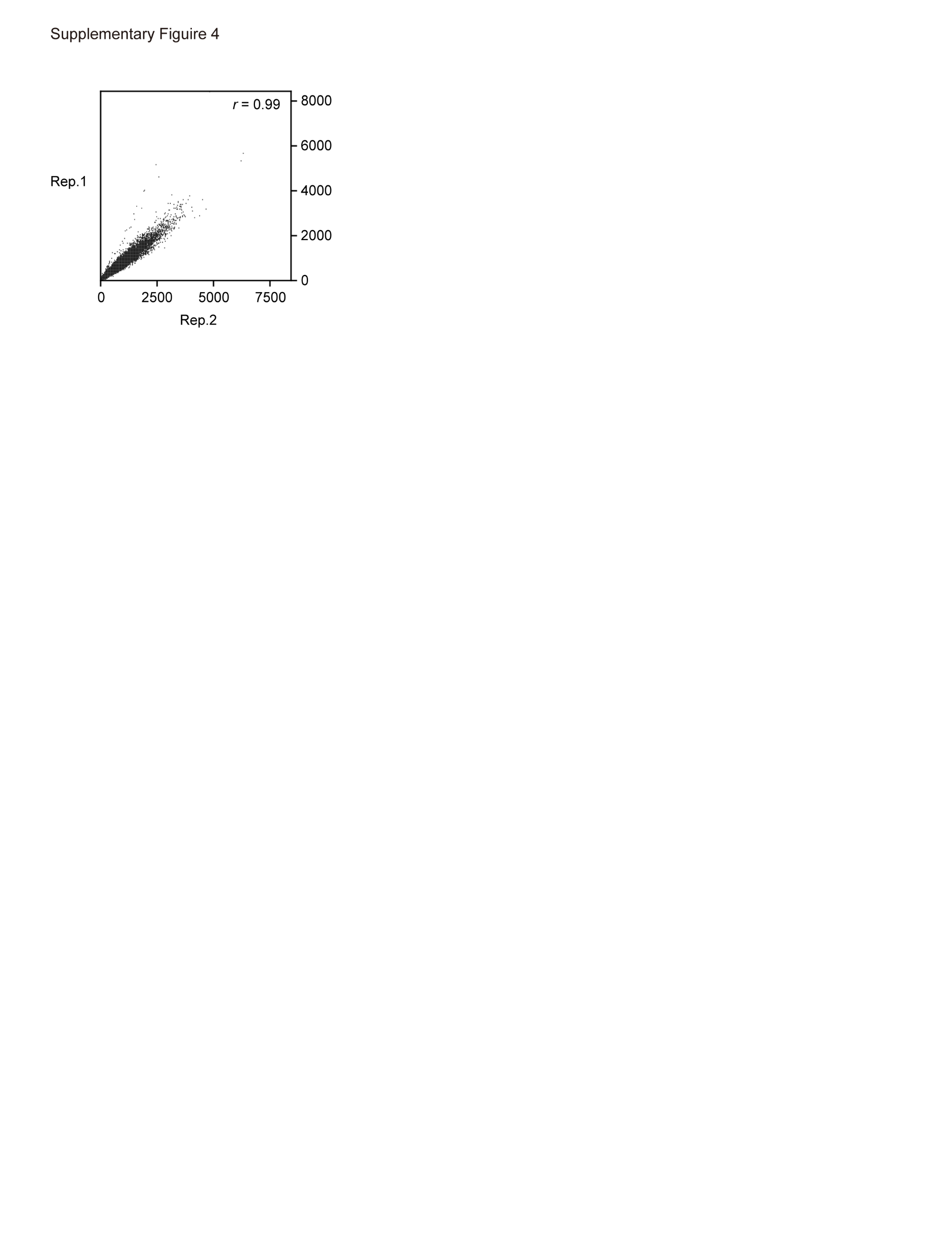


## **Supplementary Figure S4.** Assessment of neuron-specific ChIP-seq reproducibility

Scatter plot shows the correlation between technical replicates of neuron-specific ChIP-seq. The Pearson correlation coefficient (*r*) was calculated.


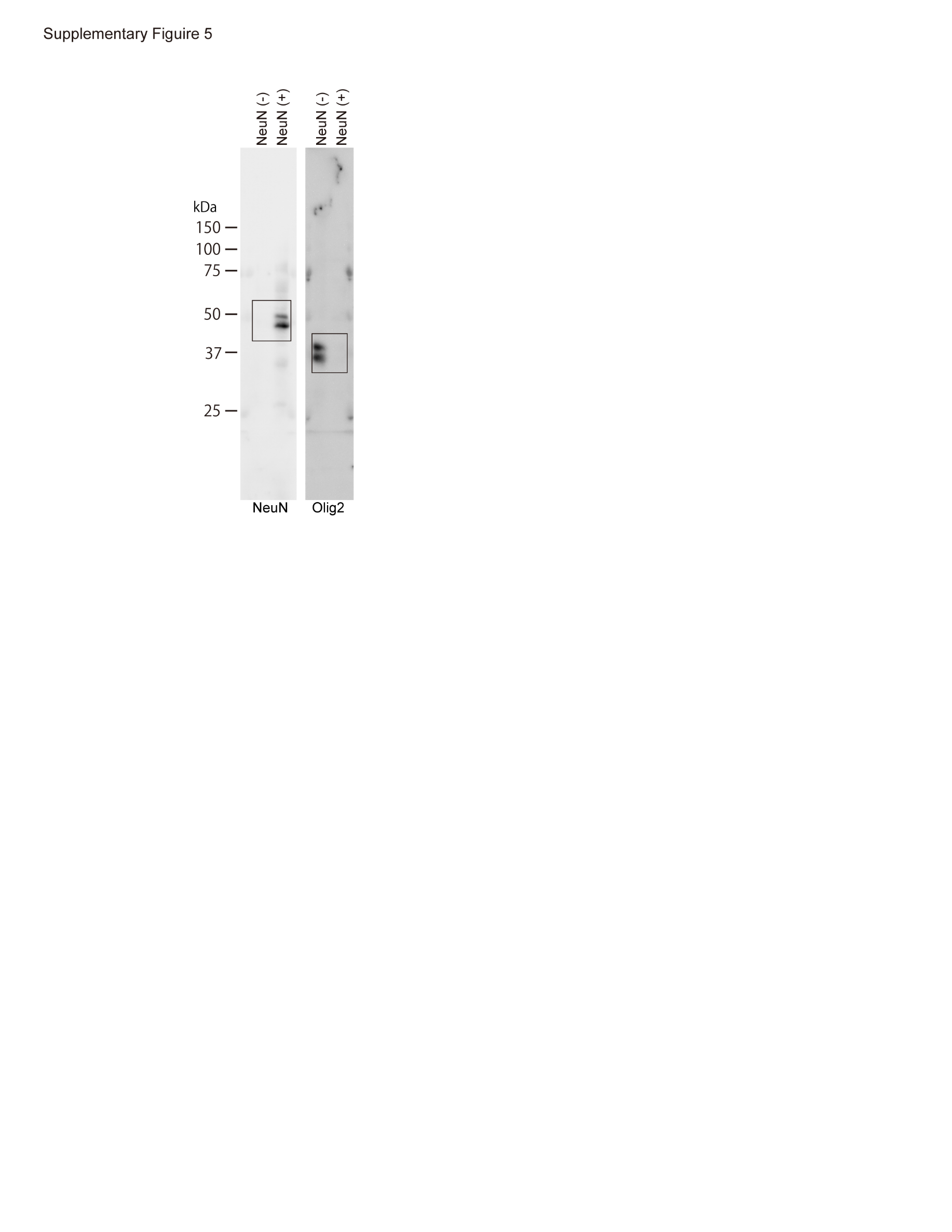


**Supplementary Figure S5.** *Uncropped Western blots of Supplementary Figure 1b*

Original full length blot of supplementary figure 1b. boxes indicated areas shown in the figure.

## **Supplementary Table S1.** Demographic features of the brain samples for ChIP-seq

| Sample Number | Age | Sex | Cause of death |
| --- | --- | --- | --- |
| 1 | 70 | Female | POEMS syndrome |
| 2 | 25 | Female | Accident |
| 3 | 82 | Female | Pneumonia |

## **Supplementary Table S2.** Demographic features of the brain samples for qPCR

| Sample Number | Age | Sex | Cause of death and medical history |
| --- | --- | --- | --- |
| 1 | 75 | Female | Liver cirrhosis |
| 2 | 75 | Male | Pneumonia |
| 3 | 76 | Female | Cause of death unspecified brain bank donor |
| 4 | 76 | Male | Lung cancer |
| 5 | 77 | Female | Depression |
| 6 | 77 | Female | Lung cancer |
| 7 | 78 | Male | Sepsis, diabetes myelitis |
| 8 | 78 | Male | COPD |
| 9 | 80 | Male | Respiratory failure, Myeloproliferative disorder |
| 10 | 80 | Male | Pneumonia, Heart failure |
| 11 | 81 | Male | Chronic heart failure |
| 12 | 81 | Male | Cause of death unspecified brain bank donor |
| 13 | 82 | Female | Lung cancer |
| 14 | 82 | Female | Pneumonia |
| 15 | 82 | Female | Multiple myeloma |
| 16 | 82 | Male | COPD |
| 17 | 83 | Male | Acute myocardial infarction |
| 18 | 84 | Female | Cause of death unspecified brain bank donor |
| 19 | 84 | Male | Pneumonia |

## **Supplementary Table S3.** The sequence of qPCR primers

The Primer sequences used in qPCR.

| Primer | Sequence (5’ – 3’) |
| --- | --- |
| GAPDH_F | GTGTCCTGCTGCCCACAGT |
| GAPDH_R | CTGAGATTGGCCCGATGGGA |
| GRIN2B_F | GCCTCCTCCACTAACGCTCC |
| GRIN2B_R | GAGGTTAGTGGCTGGAATAGA |
| HBB_F | GAAGTCCAACTCCTAAGCCA |
| HBB_R | GGTCTAAGTGATGACAGCCG |
| SYN3_F | CACGCCGATCTGTCTGTC |
| SYN3_R | CGCACCACGGTGTCT |
| BDNF_F | GTCCAGCTGATTGGTGGCTC |
| BDNF_R | GCTGGAGGCGGTGGAGAAGAA |
| ERMN_F | CCATGGATGAGTTGAGCAGCAT |
| ERMN_R | CCAGTCTGGAAGGTGCACT |
| OLIG2_F | CGCATCCAGAGTAAGTGTCC |
| OLIG2_R | CACAGCCGAGTTGAGGAG |
